# Dynamic expression of ancient and novel molluscan shell genes during ecological transitions

**DOI:** 10.1186/1471-2148-7-160

**Published:** 2007-09-10

**Authors:** Daniel J Jackson, Gert Wörheide, Bernard M Degnan

**Affiliations:** 1 School of Integrative Biology, University of Queensland, Brisbane Queensland 4072, Australia; 2 Department of Geobiology, Geoscience Centre, University of Göttingen, Goldschmidtstr.3, 37077, Göttingen, Germany

## Abstract

**Background:**

The Mollusca constitute one of the most morphologically and ecologically diverse metazoan phyla, occupying a wide range of marine, terrestrial and freshwater habitats. The evolutionary success of the molluscs can in part be attributed to the evolvability of the external shell. Typically, the shell first forms during embryonic and larval development, changing dramatically in shape, colour and mineralogical composition as development and maturation proceeds. Major developmental transitions in shell morphology often correlate with ecological transitions (e.g. from a planktonic to benthic existence at metamorphosis). While the genes involved in molluscan biomineralisation are beginning to be identified, there is little understanding of how these are developmentally regulated, or if the same genes are operational at different stages of the mollusc's life.

**Results:**

Here we relate the developmental expression of nine genes in the tissue responsible for shell production – the mantle – to ecological transitions that occur during the lifetime of the tropical abalone *Haliotis asinina *(Vetigastropoda). Four of these genes encode evolutionarily ancient proteins, while four others encode secreted proteins with little or no identity to known proteins. Another gene has been previously described from the mantle of another haliotid vetigastropod. All nine genes display dynamic spatial and temporal expression profiles within the larval shell field and juvenile mantle.

**Conclusion:**

These expression data reflect the regulatory complexity that underlies molluscan shell construction from larval stages to adulthood, and serves to highlight the different ecological demands placed on each stage. The use of both ancient and novel genes in all stages of shell construction also suggest that a core set of shell-making genes was provided by a shared metazoan ancestor, which has been elaborated upon to produce the range of molluscan shell types we see today.

## Background

The evolutionary success of certain major metazoan groups, such as the Mollusca, Bryozoa, Scleractinia, Echinodermata and Crustacea partly can be attributed to an ability to assemble a wide diversity of mineralised structures [1,2]. This capacity, in combination with environmental changes at the end of the Proterozoic [3,4], has been proposed as one of the biological characters that aided the Cambrian radiation [5]. However, it is unknown if the genetic programming directing the biofabrication of calcified and other mineralised structures in disparate animals is homologous [5]. This is because the molecular and cellular mechanisms underlying metazoan biomineralisation remain largely unknown (however see [6] for an example of a highly conserved biomineralisation gene). Further complicating this analysis is the fact that many animals with calcified skeletons produce different types of skeletons at different times in their lives, with marked changes in skeletal form, mineralogy and function often accompanying ecological transitions [7]. For example, most marine invertebrates have pelagobenthic life cycles, where metamorphosis of the microscopic larva into a benthic juvenile dramatically changes the ecology and body plan of the animal. This major ecological and morphological transition often includes a dramatic change in the form and function of the molluscan shell. It is currently unknown if ontogenetic changes in skeletal construction are the result of the expression of different batteries of biomineralisation genes, i.e. are discrete genetic networks required for larval shell formation versus adult shell formation?

All shell bearing molluscs employ a homologous organ (the mantle) to construct their shells in a way that permits an amazing phenotypic diversity [8]. The mantle consists of a variety of cell types that are directly responsible for synthesis of the shell via the secretion of an organic matrix that is able to initiate and regulate the cell-autonomous assembly of CaCO_3 _crystals [9]. Once initiated upon synthetic substrates *in vitro*, ordered CaCO_3 _crystal growth can reflect that observed in vivo [10,11]. A number of proteins implicated in the calcification process have been identified from the shells of mature animals including oysters [12-14], mussels [15-17] and abalone [11,18-20]. Despite the formulation of detailed hypotheses regarding the molecular basis of molluscan shell formation [21-24], these do not account for the initiation of biomineralisation or changes in shell construction during a mollusc's life [see [25] for a review of the mineralogical transitions that occur in larval forms]. Currently, only a handful of genes are known to play a role in the larval stages of biomineralisation [26-30], with all of these encoding molecules that either define biomineralising cells, the boundaries between biomineralising and non-biomineralising fields, or act to regulate the expression of downstream actuators of the biomineralisation process.

Here, we investigate the ontogenetic expression of a suite of genes expressed in shell forming cells and tissues in the tropical abalone *Haliotis asinina*. The juvenile mantle of *H. asinina *expresses a diverse set of genes, a large proportion of which are evolutionarily novel, are predicted to be secreted and are likely to be directly involved in shell synthesis [31]. The regionalised structure of the juvenile mantle and shell also allows inferences to be made regarding gene function [31]. Here we describe the developmental expression of nine mantle genes during the life of *H. asinina*, specifically testing if ontogenetic changes in gene expression correlate with ecological and morphological transitions. These genes are all expressed in dynamic patterns in shell forming cells and tissues, revealing the complexity of the genetic network underlying molluscan skeletogenesis. These results serve to highlight the interplay between ecology, evolution and development that has shaped the diversity of molluscan shells we see today.

## Results

### Shell ontogeny

**Figure 1:**
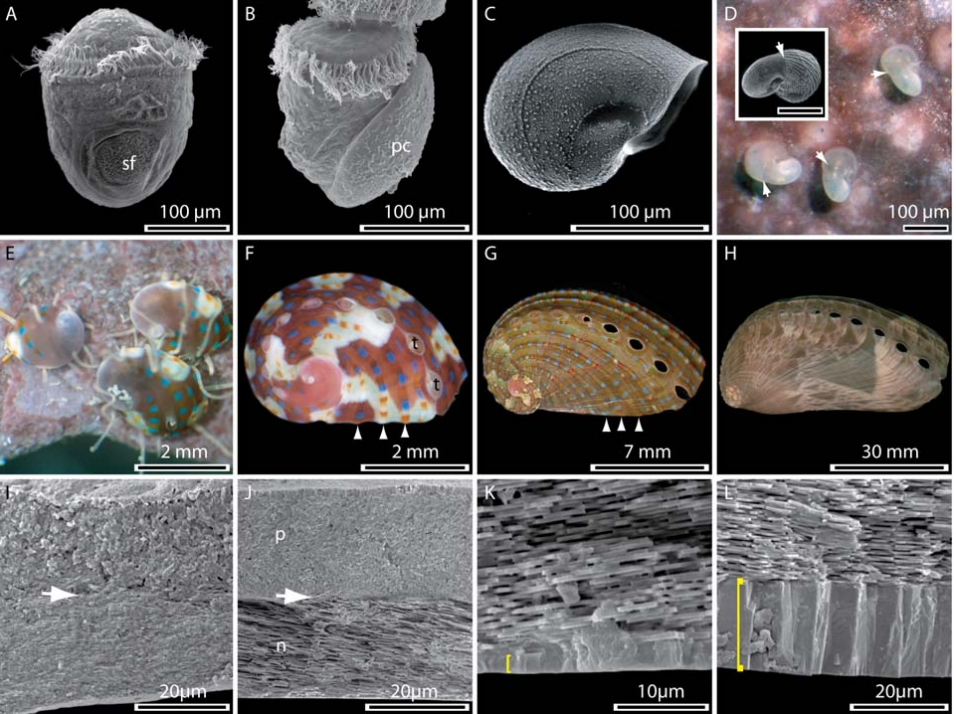
Major transitions during shell development of the tropical abalone *Haliotis asinina*. (A) A newly hatched trochophore larva 9 h post fertilisation (hpf) during the initial stages of biomineralisation; the shell field (sf) is evident. (B) The calcified protoconch (pc) is present by 11 hpf. (C) The completed larval shell displays fine sculpturing and markings indicative of a high degree of control over the biomineralisation process from an early age. (D) Newly settled postlarvae on coralline algal surface. An abrupt transition in shell morphology accompanies metamorphosis (white arrows). This initial postlarval shell is unpigmented, and displays a rippled texture (inset). (E) 1–2 month old juveniles have developed a pigmented shell that is initially a uniform maroon, but soon develops a series of blue and orange dots and cream and maroon fields. (F) Animals with a shell size of approximately 1 – 10 mm maintain this complexity in pigmentation which follows a simple set of rules: blue dots occur against a maroon background, orange dots against a cream background. Tremata (t) and ridges (arrowheads) have also developed. (G) Animals with a shell size larger than approximately 10 mm gradually loose the underlying swaths of maroon and cream pigmentation, but maintain the expression of blue and orange dots on the now prominent ridges (arrowheads). (H) The shells of sexually mature animals no longer possess blue and orange dots and the ridges present in smaller shells are less prominent. A pattern of tan – brown triangles of varying intensity now patterns the shell. (I) A cross section from the shell of a 1 mm juvenile. The ordered aragonitic tablets characteristic of later stages are not present, but a transition in crystal morphologies is evident (arrow). (J). Cross section through the shell of a 5 mm juvenile. A relatively thin layer of nacre (n) composed of ordered aragonitic tablets is overlaid by the prismatic layer (p). (K) A cross section through the shell of a 20 mm animal reveals a new structural layer of CaCO_3 _that has been added to the ventral most region of the shell (yellow bracket). (L) The thickness of this ventral most layer continues to increase and in 100 mm animals is approximately 20 μm thick.

Several major transitions in shell pattern and morphology can be observed during the life of *Haliotis asinina*. The initial differentiation of biomineralising cells is likely to include a localised thickening of the dorsal ectoderm followed by an invagination of cells to form the shell gland [32]. The shell gland then evaginates to form the shell field (Fig. 1A) which expands through mitotic divisions to direct the precipitation of calcium carbonate (CaCO_3_) via the secretion of organic molecules. In this way the larval shell (protoconch) is formed (Fig. 1B and 1C). The construction of the haliotid protoconch is complete following torsion (Fig. 1C), and remains developmentally inert until the animal contacts a specific cue that initiates the process of metamorphosis. [33,34]. The transition from protoconch to teleoconch (juvenile/adult shell) is clearly visible at metamorphosis (Fig. 1D), and suggests the action of a different biomineralising secretome. The early postlarval shell is more robust and opaque than the larval shell but has no pigmentation. Juvenile *H. asinina *begin to develop a complex colouration in the shell several weeks after metamorphosis (Fig. 1E and 1F). This pattern is gradually lost with growth as the shell becomes thicker and more elongate (Fig. 1G and 1H). These large scale morphological changes are accompanied by mineralogical and crystallographic changes (Fig. 1I–L ). Well defined tablets of nacre are present in shells larger than approximately 5 mm (Fig. 1J–L) which are absent or poorly resolved in shells 1 mm or less (Fig. 1I). In larger shells, a ventral cap of CaCO_3 _that underlies the tablets of aragonitic nacre continues to thicken (Fig. 1K and 1L).

### Temporal expression of biomineralising genes

**Figure 2:**
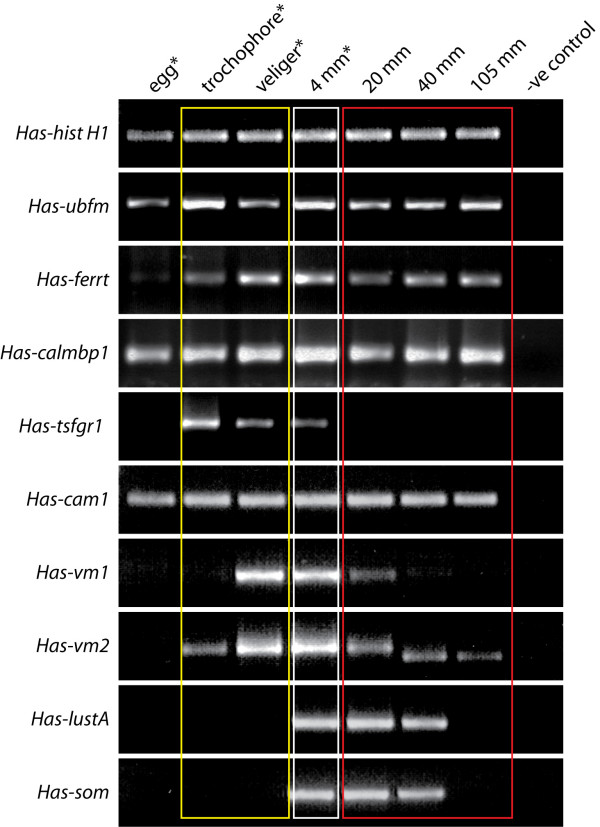
RT-PCR analysis of the relative expression levels of 9 genes expressed in shell forming cells and tissues of *H. asinina*. Histone H1 acted as a control to ensure equal cDNA synthesis efficiency and template loading into the RT-PCR reaction. Coloured rectangles group periods in development that correspond to the occupation of distinct habitats. The yellow rectangle groups planktonic larval stages. The white rectangle indicates animals of a size that occupy CCA dominated habitats. The red rectangle groups juvenile and adult stages that occupy the undersides of coral bommies. Gene abbreviations are as follows: Has-histH1, *Haliotis asinina *histone H1; Has-ubfm1, *Haliotis asinina *ubiquitin fold modifier 1; Has-ferrt, *Haliotis asinina *ferritin; Has-calmbp1, *Haliotis asinina *calcium binding protein 1; Has-tsfgr1, *Haliotis asinina *trochophore shell field glycine rich 1; Has-cam1, *Haliotis asinina *calmodulin 1; Has-vm1, *Haliotis asinina *veliger mantle 1; Has-vm2, *Haliotis asinina *veliger mantle 2; Has-lustA, *Haliotis asinina *lustrin A; Has-som, *Haliotis asinina *sometsuke. * indicates that RNA was extracted from whole animals rather than biomineralising tissues specifically.

We have isolated nine genes that are expressed in cells at the anterior edge of the mantle in the region responsible for the construction of the tropical abalone shell (see Methods and [28]). The temporal expression profiles of some of these genes coincide with ecological transitions that occur during development (Fig. 2). *Has-ubfm*, *Has-calmbp1*, and *Has-cam1 *transcripts are maternally provided to the egg, and appear to be constitutively expressed during development. *Has-ubfm*, *Has-ferrt*, *Has-calmbp1 *and *Has-cam1 *are present during embryogenesis, with detectable levels of transcripts present from the trochophore stage onwards and in all mantle biopsies surveyed. In contrast, *Has-tsfgr1*, *Has-vm1*, *Has-vm2*, *Has-lustA *and *Has-Som *are transiently expressed during the life of *H. asinina*. *Has-tsfgr1 *is highly expressed in larval stages followed by a down-regulation in 4 mm animals and a subsequent lack of expression in larger animals. *Has-vm1 *is highly expressed in veligers and juveniles ≦ 4 mm, but is absent in the mantle tissue of animals larger than 20 mm. *Has-vm2 *displays a strong up-regulation from trochophore to veliger stages, followed by a subsequent down-regulation in 20 mm animals. A smaller RT-PCR product is detected in 40 and 105 mm animals, suggesting the presence of an alternatively spliced transcript. *Has-lustA *and *Has-Som *transcripts are only detected in the mantle tissue of animals larger than 4 mm.

### Gene characterisation and spatial expression

#### Has-ubfm [DW986191]

**Figure 3:**
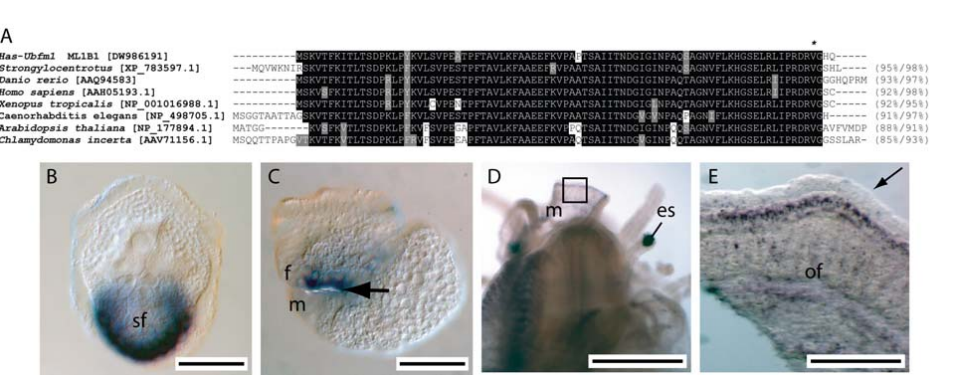
Sequence and expression analysis of *Has-ubfm*. (A) Alignment of Has-ubfm to other ubiquitin-like fold modifiers. A conserved glycine residue that is exposed following C-terminal processing and is necessary for conjugation to various target molecules is indicated by *. Sequences are followed by the percentage of sites sharing identity and biochemical similarity. Positions shaded black indicate cases where more than 50% of the residues are identical, and grey where biochemical similarity is shared with the consensus residue. GenBank accession numbers are in brackets. (B) Expression of *Has-ubfm *is restricted to the expanding edge of the shell field (sf) of trochophore larvae. (C) In pre-torsional veligers expression is associated with the foot (f) primordia. The light refractory operculum is indicated (arrow). The developing mantle (m) lies immediately adjacent to this structure. (D) Expression of *Has-ubfm *within 5 mm juveniles occurs diffusely throughout the outer fold of the mantle (m). The eye spot (es) is indicated. (E) A magnified view of the boxed region in D reveals *Has-Ubfm *expression along the anterior edge of the outer fold (of) of the mantle; the ventral-most inner fold is indicated (arrow).

Based on sequence alignments with representative GenBank sequences *Has-ubfm *encodes an open reading frame (ORF) that shares significant similarity with ubiquitin-like fold modifying proteins from various organisms (Fig. 3A). The putative full length 85 residue abalone protein possess the conserved glycine residue that is exposed following C-terminal processing required for conjugation to intracellular targets [35]. *Has-ubfm *transcripts are restricted to ectodermal cells of the expanding shell field of trochophore larvae (Fig. 3B). Expression within pre-torsional veligers is restricted to the foot primordia, and immediately adjacent to the newly forming operculum (Fig. 3C arrow), suggesting that *Has-ubfm *is necessary for the construction of this extracellular structure and the larval shell. Competent veligers do not express *Has-ubfm *within the mantle tissue but instead expression is restricted to a pair of endodermal cells associated with the digestive gland (data not shown). Following metamorphosis, *Has-ubfm *is expressed within various tissues of 1–2 mm juveniles including cells of the outer mantle fold (Fig. 3D and 3E) with a relatively high concentration of transcripts in cells along the anterior edge of the outer fold (Fig. 3E).

#### Has-ferrt [DW986406]

**Figure 4:**
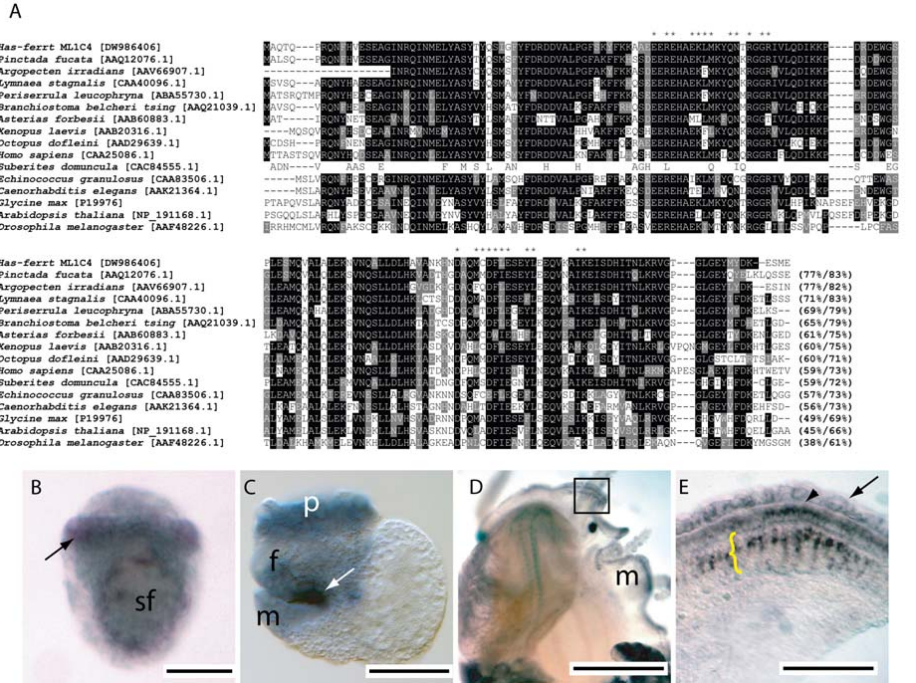
Sequence and expression analysis of *Has-ferrt*. (A) Has-ferrt shares significant similarity with members of the ferritin family. Iron binding motifs are indicated by *. Sequences are followed by the percentage of sites sharing identity and biochemical similarity. Positions shaded black indicate cases where more than 50% of the residues are identical, and grey where biochemical similarity is shared with the consensus residue. GenBank accession numbers are in brackets. (B). Expression of *Has-ferrt *within trochophore larvae is located to the shell field (sf) and prototroch (arrow). (C) Pre-torsional veligers express *Has-ferrt *in operculum forming cells of the foot (f), the prototroch (p) and mantle (m). Cells expressing *Has-ferrt *intensely within foot primordia cells immediately adjacent to the mantle where operculum formation takes place are indicated by a white arrow. (D) *Has-ferrt *expression within 1 mm juveniles is located within the mantle (m). (E) A magnified view of the boxed region in D reveals *Has-ferrt *expression restricted to the outer fold (yellow bracket), the anterior edge of the outer fold (arrowhead) and diffusely within the inner fold (black arrow).

Alignment of the derived amino acid sequence of *Has-ferrt *with ferritin proteins from a variety of metazoan taxa reveals a high degree of sequence conservation (Fig. 4A). *Has-ferrt *transcripts localise to cells of the prototroch and in ectodermal cells associated with the edge of the expanding shell field within newly hatched trochophores (Fig. 4B). Pre-torsional veligers express *Has-ferrt *diffusely within the foot primordia and the prototroch. Similar to *Has-ubfm *expression, relatively strong expression in the posterior region of the foot suggest a role for ferritin in construction of the operculum (Fig. 4C). Following metamorphosis a complex pattern of *Has-ferrt *expression is detected within the inner and outer mantle fold of 1–2 mm juveniles (Fig. 4D and 4E).

#### Has-calmbp1 [DW986217]

**Figure 5:**
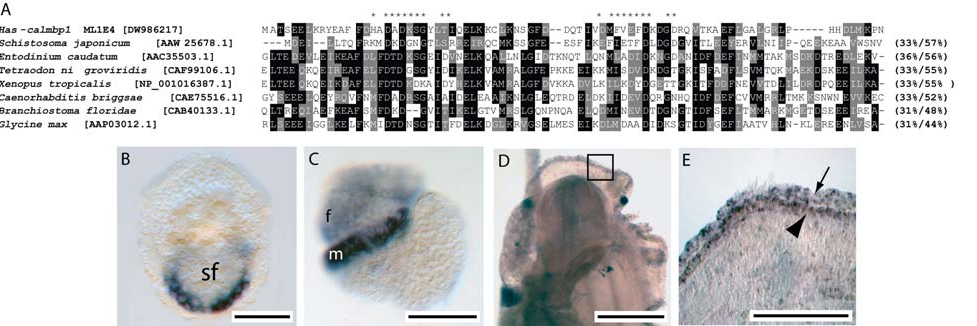
Sequence and expression analysis of *Has-calmbp1*. (A) Has-calmbp1 shares similarity with calcium binding EF proteins from various taxa. EF hand residues identified by Prosite are indicated by *. Sequences are followed by the percentage of sites sharing identity and biochemical similarity. Positions shaded black indicate cases where more than 50% of the residues are identical, and grey where biochemical similarity is shared with the consensus residue. GenBank accession numbers are in brackets. (B) Expression of *Has-calmbp1 *is restricted to the shell field (sf) of trochophores. (C) Pre-torsional veligers also express *Has-calmbp1 *in the mantle. (D) *Has-calmbp1 *is expressed diffusely throughout the mantle of 1 mm juveniles. (E) A magnified view of the boxed region in D reveals *Has-*calmbp1 transcripts are concentrated along the anterior edge of the outer fold (arrowhead) and the inner mantle fold (arrow) (E).

*Has-calmbp1 *encodes a protein with sequence similarity to proteins with Ca^2+ ^binding sites (Fig. 5A). Spatial expression of *Has-calmbp1 *within trochophores is restricted to ectodermal cells associated with the lateral edges of the expanding shell field (Fig. 5B). Prior to torsion, mantle cells express *Has-calmbp1 *intensely (Fig. 5C) in contrast to the subsequent lack of expression within the mantle of competent veligers (data not shown). Following metamorphosis *Has-calmbp1 *is expressed continuously along the length of the mantle margin (Fig. 5D and 5E).

#### Has-tsfgr1 [DW986319]

**Figure 6:**
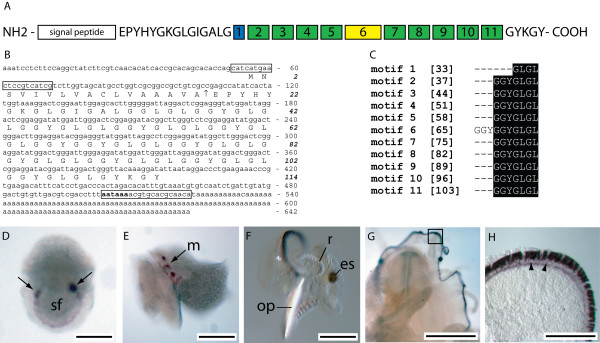
Sequence and expression analysis of *Has-tsfgr1*. (A) A schematic representation of the conceptually derived Has-tsfgr1 protein. A putative signal peptide followed by 15 residues constitute the amino end of the protein. 11 glycine rich domains then follow, predominantly containing the motif 'GGYGLGL'. Boxes of the same colour indicate identical sequence motifs. 5 residues at the carboxyl terminus (GYKGY) may share functional homology with the Shematrin proteins. (B) The *Has-tsfgr1 *transcript is 642 bp long and contains a polyadenylation signal (bold). Primers used to assess temporal expression are boxed, and the predicted signal sequence cleavage site is indicate by ↑. (C) An alignment of the repetitive domains of Has-tsfgr1 reveal the high degree of sequence conservation between motifs. (D) *Has-tsfgr1 *transcripts are initially detected in trochophore larvae within the expanding shell field (sf) with relatively strong expression in an anterior pair of cells (arrows). (E) Early post-torsional veligers express *Has-tsfgr1 *in a triplet of cells spread along the developing mantle edge on the left side of the animal. This lateral view presents the animal with anterior to the left. (F) Competent veligers express *Has-tsfgr1 *continuously along the mantle edge. The eyespot (es), radula (r) and refractive operculum (op) are indicated. (G) Intense expression of *Has-tsfgr1 *is detected within the mantle of 1 mm juveniles. (H) A magnified view of the boxed region in G reveals that this expression is restricted to the anterior edge of the outer mantle fold. The apical expression of *Has-tsfgr1 *within these cells is clearly visible (nuclei indicated by arrowheads).

Following an EST screen for genes expressed in the mantle tissue of juvenile abalone *Has-tsfgr1 *was identified as possessing an ORF suggestive of a role in biomineralisation (Fig. 6A). The 642 bp transcript of *Has-tsfgr1 *encodes a putative 114 amino acid ORF comprised of a 17 residue signal sequence and a mature protein consisting of glycine, leucine and tyrosine repeats (Fig. 6A–C). Has-tsfgr1 shares some similarity with glycine rich sequences within public sequence databases such as spidroin [36,37], however the repetitive nature and high glycine content of this protein make it difficult to assign any evolutionary homology. WMISH results indicate that *Has-tsfgr1 *is restricted to the expanding edge of the shell field of newly hatched trochophores, with the 2 anterior-most cells of the shell field expressing this transcript strongly (Fig. 6D). Early post-torsional veligers express *Has-tsfgr1 *in a set of 3 mantle cells on the left side of the animal (Fig. 6E). Competent veligers localise *Has-tsfgr1 *transcripts relatively consistently along the mantle edge (Fig. 6F). Following metamorphosis,*Has-tsfgr1 *expression is restricted to the anterior edge of the outer mantle fold mantle in 2 mm animals (Fig. 6G and 6H).

#### Has-cam1 [DW986371]

**Figure 7:**
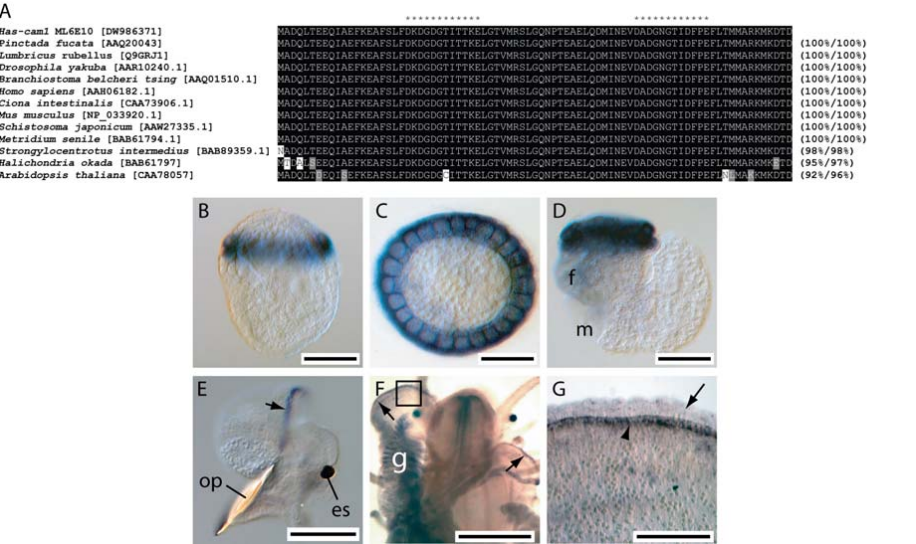
Sequence and expression analysis of *Has-cam1*. (A). Has-cam1 shares significant similarity with calmodulin proteins from a range of taxa. Calcium binding EF hand motifs identified by Prosite are indicated by *. Sequences are followed by the percentage of sites sharing identity and biochemical similarity. Positions shaded black indicate cases where more than 50% of the residues are identical, and grey where biochemical similarity is shared with the consensus residue. GenBank accession numbers are in brackets. (B) *Has-cam1 *transcripts are located in the prototroch of trochophore larva. lateral view. (C) Apical view of trochophore larva. (D) Pre-torsional veligers also express *Has-cam1 *in the prototroch. The foot (f) and mantle (m) primordia are indicated. (E) Competent veligers express *Has-cam1 *in the mantle (arrow). The operculum (op) and eye spot (es) are indicated. (F) 1 mm juveniles express *Has-cam1 *within the gills (g) and the mantle (arrows). (G) Magnified view of the boxed region in F. *Has-cam1 *transcripts are restricted to the anterior edge of the outer mantle fold (arrowhead). The inner mantle fold is also visible (arrow).

The *Has-cam1 *derived protein sequence displays a high degree of sequence similarity to calmodulin proteins across a broad range of taxa (Fig. 7A). Trochophores express *Has-cam1 *predominantly within the ciliated cells of the prototroch (Fig. 7B and 7C). This pattern of expression is maintained until prior to torsion (Fig. 7D). Competent veligers express *Has-cam1 *within the mantle (7E). Following metamorphosis *Has-cam1 *transcripts are detected within the gills of 1–2 mm juveniles and in the anterior edge of the outer mantle fold (Fig. 7F and 7G).

#### Has-vm1 [DQ328317]

**Figure 8:**
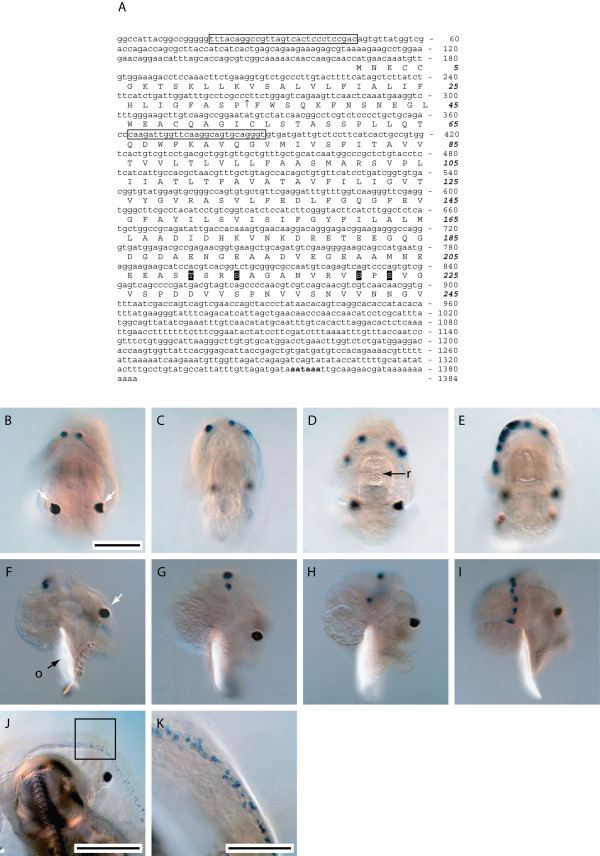
Sequence and expression analysis of *Has-vm1*. The putative full length *Has-vm1 *transcript encodes an ORF of 245 amino acid residues. A putative signal sequence (↑), glycosylation sites (highlighted black) and polyadenylation signal (bold) are indicated. Primers used to assess temporal expression are boxed. (B – E) anterior views. (B) *Has-vm1 *expression begins approximately 60 hours post fertilisation (hpf) in a pair of cells located in the dorsal mantle edge of the veliger larva. Eyespots are indicated by white arrows. (C) By 72 hpf expression has expanded to 3 cells in the dorsal mantle edge. (D). An 84 hpf larva with 4 *Has-vm1 *positive mantle edge cells. The chitinised teeth of the radula (r) are visible. (E). By 132 hpf expression of *Has-vm1 *forms an asymmetrical, left sided pattern in 9–10 cells along the mantle edge, and is maintained in this way until metamorphosis is induced. (F-I) Corresponding right side lateral views of the larvae depicted in B-E. (J) Following metamorphosis expression of *Has-vm1 *proliferates along the mantle edge of 1 mm juveniles; (K) expanded boxed section in J. The discrete, punctate expression observed in competent veligers has now become a continuous line of cells along the entire length of the mantle edge.

The putative full length *Has-vm1 *transcript of 1384 bp encodes a conceptually derived proprotein of 319 residues that shares no significant similarity with proteins or nucleotide sequences within public databases, including whole genome shotgun traces of the patellogastropod *Lottia scutum *[38]. The derived protein possesses a signal sequence of 17 residues suggesting this protein is secreted from the cell. One threonine and three serine residues are predicted to be glycosylated (Fig. 8A). Spatial expression of *Has-vm1 *in competent veligers is highly punctate and is restricted to the mantle (Fig. 8B–I). *Has-vm1 *expression progresses from a pair of dorsal cells (Fig. 8B and 8F), to more laterally positioned cells (Fig. 8C,D,G and 8H), until an asymmetrical, right-biased distribution of cells is achieved (Fig. 8E and 8I). This pattern is maintained until metamorphosis is induced. Following metamorphosis *Has-vm1 *transcripts are located in a dispersed but continuous pattern along the entire length of the mantle margin of 1 mm juvenile animals (Fig. 8J and 8K).

#### Has-vm2 [DQ298397]

**Figure 9:**
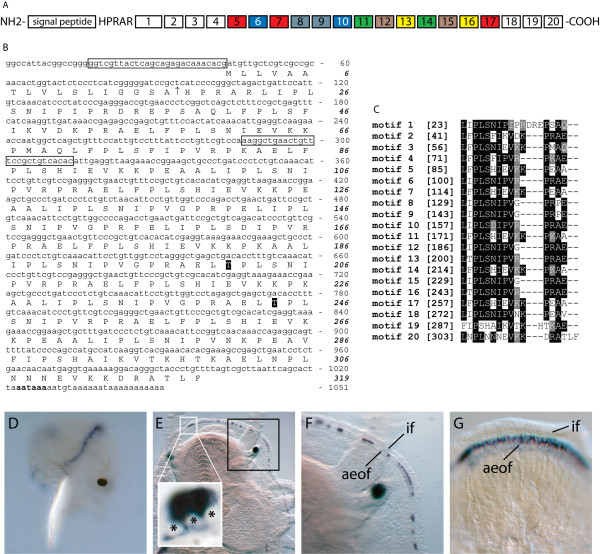
Sequence and expression analysis of *Has-vm2*. (A) A schematic representation of the conceptually derived Has-vm2 protein. A putative signal peptide followed by 5 residues precede 20 proline-rich motifs. Identical motifs are indicated by boxes of the same colour. (B) The putative full length 1051 bp *Has-vm2 *transcript encodes an ORF of 319 residues, with two predicted glycosylated threonine residues. Primers used to assess temporal expression are boxed. (C) When the 20 proline-rich motifs are aligned, conserved Leu, Pro, Ser, Ile, Val, Glu and positively charged (Arg and Lys) residues are revealed. Residues are shaded black where more than 50% of the residues are identical, and grey where biochemical similarity is shared with the consensus residue. Numbers to the left indicate amino acid position within the proprotein. (D) *Has-vm2 *expression is initially detected in a continuous pattern along the mantle of competent veligers. (E) Expression of *Has-vm2 *within the mantle of 1 mm juveniles is restricted to the anterior edge of the outer mantle fold. A characteristic triplet of cells (inset) is detected within individuals of this size making the basal nuclei visible (*). (F) A magnified view of the boxed region in E. A characteristic dot and dash expression of *Has-vm2 *within the anterior edge of the outer fold (aeof) is present at this stage of development. The inner fold (if) and anterior edge of the outer fold (aeof) of the mantle is indicated. (G) The dot and dash expression of *Has-vm2 *within 1 mm juveniles eventually merges to become a continuous band of expression along the anterior edge of the outer fold (aeof) of 5 mm juveniles.

*Has-vm2 *encodes an ORF with proline-rich repeats. Comparison of Has-vm2 with the public sequence databases reveals a range of proteins that also possess repeated proline residues. As is the case for Has-tsfgr1, it is difficult to infer whether this is due to sequence homology, or whether it is the result of a functional convergence on a motif that provides a particular structure and/or function. Alignment of the 20 proline-rich domains within Has-vm2 highlights the repetitive nature of this protein (Fig. 9A and 9C). The mature protein is also predicted to possess a signal sequence and two glycosylation sites (Fig. 9B). Although RT-PCR results indicate *Has-vm2 *is expressed in trochophores (Fig. 2), repeated attempts to localise expression via WMISH failed to detect any signal at this stage of development, suggesting it is expressed at a low level at this stage. Expression of *Has-vm2 *in competent veligers is predominantly restricted to mantle cells (Fig. 9D), and is relatively constant along the length of the mantle margin (cf. *Has-vm1*). Following metamorphosis, *Has-vm2 *expression becomes characteristically punctate (Fig. 9E and 9F) with some consistency to this pattern between individuals suggesting that the precise pattern of punctuation may be an important functional feature of the protein, e.g. a characteristic triplet of cells expressing *Has-vm2 *was observed among all 1 – 2 mm individuals assayed (Fig. 9E inset). This dot and dash pattern is replaced in larger juveniles (5 mm) by a continuous line of expression in the anterior edge of the outer fold of the mantle (Fig. 9G).

#### Has-lustA [DQ298402]

**Figure 10:**
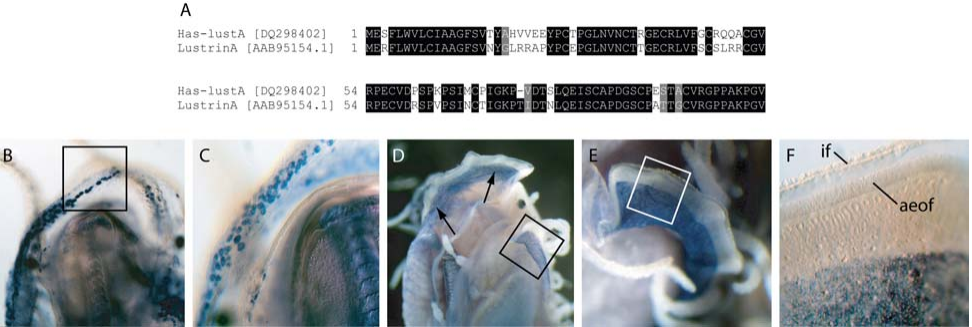
Sequence and expression analysis of *Has-lustA*. An alignment of Has-lustA with Lustrin A from *Haliotis rufescens *illustrates the high degree of sequence conservation. Positions shaded black indicate cases where more than 50% of the residues are identical, and grey where biochemical similarity is shared with the consensus residue. GenBank accession numbers are in brackets. (B) Spatial expression of *Has-LustA *in 1 mm juveniles begins with a population of cells along the left side of the animal. (C) Magnified view of the boxed region in B. At this stage of development individual, non-confluent cells expressing *Has-lustA *are visible. (D) In 5 mm juveniles *Has-lustA *positive cells have proliferated to become densely packed in the proximal region of the outer fold. (E) Magnified view of the boxed region in D. (F) Magnified view of the boxed region in E. The anterior edge of the outer fold (aeof) and the inner fold (if) are indicated. *Has-lustA *positive cells represent the region of the mantle responsible for the deposition of nacre.

A partial fragment of Has-lustA aligns with Lustrin A from *H. rufescens *revealing a high level of sequence conservation (Fig. 10A). Spatial expression of *Has-lustA *in 1 – 2 mm animals identifies a population of cells that are initially located within the proximal region of the left mantle lobe (Fig. 10B and 10C). As development progresses, cells expressing *Has-lustA *proliferate until there is a uniform field of expression in the proximal region of both mantle lobes (Fig. 10D–F). This region is responsible for nacre synthesis [31].

#### Has-Som [DW986219]

**Figure 11:**
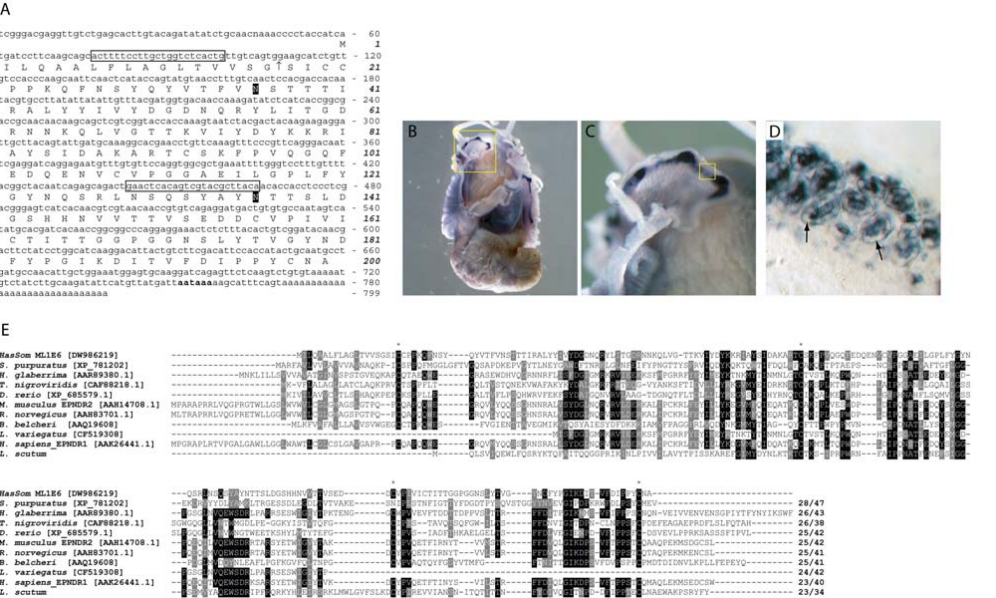
Sequence and expression analysis of *Has-Som*. The *Has-Som *transcript encodes an ORF of 200 residues. A putative signal sequence (↑), two glycosylated Asp residues (highlighted black), and a polyadenylation signal (bold) are indicated. (B) *Has-Som *expression in 4 mm juveniles is restricted to the mantle. (C) Magnified view of the boxed region in B illustrates the expression of *Has-Som *in the outer fold of the mantle. (D) Expanded view of the boxed region in C reveals the morphology of *Has-Som *expressing cells with large vacuoles (arrows). (E) An alignment of *Has-Som *with deuterostome ependymin proteins illustrates the low level of sequence conservation. The four cysteine residues required for disulfide linkages in deuterostome taxa are indicated (*). Sequences are followed by the percentage of sites sharing identity and biochemical similarity. Positions shaded black indicate cases where more than 50% of the residues are identical, and grey where biochemical similarity is shared with the consensus residue. GenBank accession numbers are in brackets.

Following an EST screen for genes involved in the process of biomineralisation [31], a consensus of 18 EST clones yielded a putative full length *Has-Som *transcript of 799 bp encoding a 200 residue proprotein (Fig. 11A). Has-Som possesses a 17 residue signal sequence and shares some primary structural features with the ependymin proteins, in particular 6 cysteine residues, 4 of which are highly conserved (Fig. 11E), and two putative N-linked glycosylation sites (Fig. 11A). *Has-Som *expression in the mantle overlaps with blue colour in the shell, strongly suggesting the gene product contributes to shell pigmentation [31]. *Has-Som *transcripts localise to cells with large vacuoles in the outer mantle fold of 5 mm juveniles (Fig 11B–D).

## Discussion

We recently have shown that the juvenile *Haliotis asinina *mantle transcriptome is rapidly evolving and extremely complex [31]. It is evident that hundreds of proteins are secreted from the gastropod mantle into the vicinity where biomineralisation occurs. These are likely to be involved in shell synthesis, presumably contributing directly to the patterning and construction of the calcified shell. The regulation and production of the abalone shell is at least an order more complex than has been inferred from a compilation of previous studies on shell matrix proteins in numerous other molluscs as acknowledged by Marin and Luquet [39]. This 'secretome' complexity, in combination with the modular organisation of the mantle into distinct territories responsible for the biofabrication of discrete shell layers [31,40], provides a foundation for the generation of diverse shell types. While we cannot yet provide functional data for any of the genes we have studied here, based on their expression profiles we can infer another level of complexity in the regulation of these shell genes at different life cycle stages. The temporal regulation of biomineralisation genes is likely to be an important factor in the production of ontogenetically discrete shell types. For *H. asinina*, ontogenetic changes in the expression of genes likely to be directly involved in the process of biomineralisation correlate with habitat and ecological transitions.

### Changes in shell structure and pattern track with *H. asinina's *ecology

*H. asinina *has a pelagobenthic life cycle that includes a minimal period of three to four days in the plankton [33,41]. The first biomineralisation events occur shortly after hatching, with the fabrication of the larval shell (protoconch) over about a 10 h period. These structures allow the veliger larva to completely retract into a protective environment and rapidly fall out of the water column. The next phase of biomineralisation does not commence until the competent veliger larva contacts an environmental cue that induces metamorphosis [41]. Postlarval shell (teleoconch) is laid down rapidly following metamorphosis with marked variation in the rate of its production between individuals. While the initial teloconch is not pigmented (Fig. 1D), it is textured and opaque such that postlarval shell growth is easily discerned from the larval shell (Fig. 1D inset). Subsequently, the teloconch rapidly develops a uniform maroon colouration similar to the crustose coralline algae (CCA) that the larva has settled upon (Fig. 1E). At about 1 mm in size further changes in the morphogenetic program of the mantle are reflected in the shell. Structurally, a pronounced series of ridges and valleys and a line of respiratory pores (tremata) have appeared (Fig. 1F). Furthermore, it is at this stage of development that the first recognisable tablets of nacre can be detected (Fig. 1J). Colourmetrically, the uniform maroon background is now interrupted by oscillations of a pale cream colour, and is punctuated by a pattern of dots (that only occur on ridges) which are blue when overlying a maroon field and orange when overlying a cream field. This shell pattern may enhance the juvenile's ability to camouflage on the heterogeneous background of the CCA they inhabit at this stage of development.

At 10 to 15 mm, this ornate colouration pattern begins to fade, with maroon and cream fields apparently blending to give a brown background. Blue and orange dots however persist on the ridges (Fig. 1G). With further growth, the ridge-valley structure fades to give rise to a smooth adult shell, with irregular brown-green triangles on a light brown background (Fig. 1H). These larger animals are nocturnal, graze amongst turf algae [42] and inhabit the undersides of boulders and coral bommies [43]. Overall, ontogenetic changes in *H. asinina *shell pigmentation and structure match changes in the habitats occupied during development.

### Differential expression of mantle genes reflect changes in shell structure

The spatial and temporal expression patterns of the nine genes investigated here reveal a complexity to the genetic networks that coordinate the deposition of larval, juvenile and adult shell. Many of the genes analysed in this study are expressed in the mantle during the production of larval, juvenile and adult shells (*Has-Ubfm, Has-ferrt, Has-calmbp1*), while others are restricted to one or two shell phases (*Has-tsfgr1, Has-cam1, Has-vm1, Has-vm2, Has-lustA, Has-Som*). While the lack of a detailed cell fate map through metamorphosis prevents conclusions from being drawn regarding cellular developmental homologies, the continuous expression of *Has-tsfgr1 *and *Has-vm1 *in cells no other than shell forming cells in both larval and postlarval stages suggests that a proportion of the postlarval mantle is derived from cells of the larval shell field.

Analyses of the expression profiles of the genes included in this study provide insight into the morphogenetic activity of shell production at different stages in the life of *H. asinina*. Genes that are continuously expressed in the mantle – *Has-ferrt*, *Has-ubfm *and *Has-calmbp1 *– are likely to play fundamental roles in biomineralisation. *Has-ubfm *encodes a highly conserved ubiquitin fold-like modifying protein [35] and is expressed in the expanding shell field suggests that specific intracellular processing of gene products is required to generate functional extracellular components of the biomineralising secretome. Two other evolutionarily conserved proteins, Has-ferrt and Has-calmbp1, are also expressed within the trochophore shell field and later in the mantle, and also are likely to be involved in intracellular events necessary for shell deposition. Iron is known to affect calcification processes in mammals [44,45], algae [46] and molluscs [13]. The high expression level of *Has-ferrt *in a range of cell types in the juvenile mantle is compatible with iron being essential for shell construction or pigmentation. Has-calmbp1 is similar to calcium dependent protein kinases, however its role in shell production remains unknown.

In contrast to these constitutively expressed genes, *Has-tsfgr1 *displays a dynamic expression profile in the shell forming tissue during development. The putative protein is composed of a set of glycine-rich repeats (over 52% Gly in the mature protein), suggesting it may possess elastomeric properties known to be important in various calcification processes [47]. The highly repetitive nature of Has-tsfgr1 also suggests that this protein may be involved in forming the organic template upon which initial CaCO_3 _nucleation occurs [48]. Recently Yano et al. [14] isolated a family of glycine rich, repetitive motif proteins (Shematrins) from a mantle cDNA library of the pearl oyster *Pinctada fucata*. The Shematrin family is currently known to encode 7 proteins with similar C-terminal motifs which terminate in a tyrosine residue and are expressed in the mantle edge, apparently localised to the prismatic layer of the mature oyster shell [14]. Interestingly, Has-tsfgr1 also possesses a C-terminal motif of 5 residues terminating in a tyrosine residue, suggesting that this feature may be of functional importance to this class of protein. Although sequence alignments of the Shematrins and Has-tsfgr1 do not reveal any close sequence homology, the high glycine content, repetitive nature and shared spatial expression suggest these proteins may play similar functional roles. Unlike *Has-ubfm*, *Has-calmbp1*, and *Has-ferrt*, which all maintain expression within juvenile and adult mantle tissue, *Has-tsfgr1 *is significantly down-regulated in the mantle tissue of >20 mm animals. This observation is compatible with different stages of shell development requiring the secretion of discrete sets of structural proteins, which act to alter the physical and mineralogical characteristics of the shell.

Two other novel genes – *Has-vm1 *and *-vm2 *– also display a dynamic temporal expression during the development of the shell. As expression of *Has-vm1 *is activated in the larval mantle after completion of the construction of the larval shell, this gene is likely to be involved in the construction of the postlarval shell following metamorphosis. This pattern of expression reveals a linkage between larval and postlarval mantles and is similar to that observed for the developmental regulator *Has-Hox4 *[30]. It also demonstrates that although larval shell synthesis has ceased, transcriptional activity in the larval mantle continues in anticipation for the next life cycle phase [49].

*Has-vm2 *is also differentially regulated during shell growth, and encodes a protein with the hallmarks of being involved in biomineralisation including a signal sequence, repetitive proline rich motifs and two putative O-linked glycosylation sites. *Has-vm2 *is also down-regulated in larger individuals, with a concomitant reduction in transcript size suggesting that alternative splicing of this gene product takes place in animals larger than 40 mm, again highlighting the different requirements for shell construction at different life cycle stages.

Three genes – *Has-cam*, *Has-lustA *and *Has-Som *– are expressed in patterns that are indicative of roles in biomineralisation during post-larval shell growth. Reflective of the various roles it plays within well studied mammalian systems including signal transduction and regulation of the cell cycle [50-53], *Has-cam1 *appears to play diverse roles during development. Highly expressed in the prototroch of trochophores, it is not until the veliger larva attains competence to metamorphose that *Has-cam1 *is detected within the mantle. This suggests that Has-cam1 is not directly involved in larval shell synthesis. In agreement with studies on bivalves [12,54]*Has-cam1 *is expressed within the gills and the outer fold of the mantle of juvenile animals. The expression pattern of *Has-lustA *supports its proposed role of binding aragonitic tablets of nacre together [18,55], and coincides with the appearance of ordered aragonitic tablets. Interestingly, *Has-lustA *is down-regulated in the mantle tissue of mature abalone of 100 mm (Fig. 2) possibly reflecting a cessation of shell growth as this is close to the maximum size of 11 cm reported for this species [42]. Has-Som has previously been shown to play a role in pigmentation of the juvenile shell [31] and is expressed in the mantle tissue of juvenile animals at the time complex colour patterning commences.

Many planktonic molluscan larvae face similar challenges during larval life and the evolution of a larval shell has clearly been a successful response to these challenges [56]. On the molecular level, it is currently unknown the degree to which construction of the molluscan protoconch is conserved. Previous studies have revealed that the expression of the engrailed transcription factor in polyplacophoran [28], gastropod [27,29,57] and scaphopod [26] representatives is restricted to cells that form boundaries between shell forming and non-shell forming ectoderm, suggesting that the regulatory mechanisms that establish shell forming structures in these clades were inherited from a common ancestor. Following metamorphosis, planktonic molluscan larvae inhabit a broad diversity of benthic ecological niches from sediments, coral reefs, deep sea hydrothermal vents and temperate rocky reefs. The shell of the tropical abalone undergoes several major transitions in morphology, mineralogy and pigmentation during its construction, each of which is adapted to suit the different habitats that larval, juvenile and adult forms occupy. These varied morphologies are the result of differential gene expression of both evolutionarily ancient and novel genes within the mantle tissue.

## Conclusion

Given the number of reports of molluscan biomineralising genes that do not share homology with any other phyla (see [39] for a review) and the data reported here, we suggest that the rapid evolution of the mantle secretome has greatly contributed to the radiation and evolutionary success of the Mollusca [31]. This study demonstrates that the regulation of these genes can be complex, with different batteries of structural genes activated in different parts of the mantle at different phases of the life cycle. We show that changes in expression correlate with changes in shell structure, colour and pattern, and that these changes map closely with ecological transitions. We propose that the regulation of this rapidly evolving mantle secretome is achieved through the action of highly conserved transcription factors and signalling molecules. Dissection of the gene regulatory networks controlling the construction of both larval and postlarval shells promises to shed light on the interplay between ecology and development on evolution of the molluscan body plan.

## Methods

### Analysis of mantle genes

The genes investigated in this study were originally identified either through (1) an expressed sequence tag (EST) screen of genes expressed in the mantle of juvenile abalone [31], (2) an EST survey of developmentally expressed genes [58] or (3) a differential display analysis of developmentally regulated genes [33]. Full length cDNA sequences for the genes used in this study were obtained using a RACE approach as described in Jackson et al. [33]. cDNA sequences were initially characterised as described in Jackson et al. [31] and classified as either having conceptually derived amino acid sequence similarity with proteins in public databases, or encoding a novel secreted protein. The presence of signal peptides was inferred using the SignalP 3.0 server [59] and glycosylation predictions were made using the NetOGlyc server [60]. Putative open reading frames (ORFs) for the evolutionarily novel and divergent genes *Has-vm1 *(*H. asinina – veliger mantle 1*), *Has-vm2 *(*veliger mantle 2*), *Has-tsfgr1 *(*trochophore shell field glycine rich 1*) and *Has-Som *(*Sometsuke*) were identified using ORF Finder [61].

For genes encoding conserved proteins (*Has-ubfm*, *ubiquitin fold modifier*; *Has-cam1*, *calmodulin*; *Has-ferrt*, *ferritin*; *Has-calmbp1*, *calcium binding protein*; *Has-lustA*, *Lustrin A*), tBLASTx and BLASTp searches were conducted against the GenBank database using default settings (*Has-Som*, *Sometsuke*, has been previously characterised [31]). Publicly available protein sequences that displayed significant similarity to *H. asinina *sequences and representing a broad taxonomic range were downloaded and aligned in ClustalX. Alignments were manually edited in MacClade. Percent identity and biochemical similarity for each sequence relative to the respective *H. asinina *sequence were calculated using the NCBI bl2seq algorithm [62].

### Animals and whole mount *in situ *hybridisation

Animals were procured from natural spawnings conducted at the Bribie Island Aquaculture Research Centre, Queensland, Australia as described in Jackson et al. [33]. Larvae and juvenile abalone were relaxed in approximately 0.3 M MgCl_2 _in FSW prior to fixation in 4% paraformaldehyde in 0.1 M 3-(N-morpholino) propane sulfonic acid pH 7.5 (MOPS), 2 mM MgSO_4_, 1 mM ethyleneglycoltetraacetic acid (EGTA) and 0.5 M sodium chloride (NaCl) for 30 min at room temperature. Fixed samples were then rinsed several times with PBS buffer plus 0.1% Tween 20 and stepped into 75% ethanol and stored at -20°C. Decalcification of the juvenile shell was achieved by incubation in a solution of 4% paraformaldehyde, 1× PBS buffer and 350 mM ethylenediaminetetraacetic acid (EDTA) for 1 – 3 h depending on shell size. The remaining periostracum and proteinaceous components of the shell were manually dissected away from the animal with fine dissecting forceps. Whole mount *in situ *hybridisation using digoxigenin-labeled riboprobes synthesised from PCR templates was performed following Giusti et al. [63] and Jackson et al. [31].

### Reverse transcriptase PCR

Total RNA was extracted from eggs, 10 h old newly hatched trochophores, 134 h old competent veligers, whole 4 mm (shell length) juveniles, 20 mm juvenile mantle tissue, 40 mm juvenile mantle tissue and 100 mm adult mantle tissue using TriReagent following the manufactures instructions. cDNA was synthesised from 1 μg of intact total RNA following Jackson et al. [33]. Relative levels of gene expression across the 7 cDNA samples were assessed by empirically determining the linear phase of PCR amplification for each gene using gene specific primers (available upon request). Briefly, each PCR was run for 20 cycles after which time 4 μl was removed. Reactions were then allowed to continue for a further 3 cycles and the process repeated until aliquots had been obtained from cycles 20 – 34. Samples were then separated on 2% agarose gels [64]. Each PCR reaction was run in duplicate with cDNA synthesised in the absence of MMLV-RT as a control for genomic DNA contamination. Histone H1 was used as a constitutively expressed housekeeping gene and as an indicator of equivalent cDNA synthesis efficiency and PCR template quality [33,65].

### Scanning electron microscopy

Nine, 10 and 11 h old trochophores were fixed in 3% glutaraldehyde in 0.1 M sodium cacodylate buffer for 30 min then washed in the same buffer prior to postfixing in 1% osmium tetroxide (OsO_4_) in 0.1 M sodium cacodylate buffer. Samples were dehydrated through a graded series of ethanol before being infiltrated and dried overnight in hexamethyldisilasane (HMDS). The soft tissue of competent veligers and newly metamorphosed post-larvae was dissolved using 2.8% v/v sodium hypochlorite for approximately 5 min. The remaining shells were then washed extensively with de-ionised water and dehydrated with 100% ethanol before mounting. All samples were mounted either on double-sided tape or Leit-C conductive carbon cement on aluminium stubs and sputter-coated with gold. Samples were viewed with an S-2300 Hitachi scanning electron microscope at 10 kV.

## Abbreviations

*Has-ubfm, Haliotis asinina *Ubiquitin fold modifier 1; *Has-ferrt, Haliotis asinina *ferritin; *Has-calmbp1, Haliotis asinina *calcium binding protein 1; *Has-tsfgr1, Haliotis asinina *trochophore shell field glycine rich 1; *Has-Cam1, Haliotis asinina *calmodulin 1; *Has-vm1, Haliotis asinina *veliger mantle 1; *Has-vm2, Haliotis asinina *veliger mantle 2; *Has-lustA, Haliotis asinina *lustrinA; *Has-Som, Haliotis asinina sometsuke*.

## Authors' contributions

DJJ contributed to the conception and design of the project, analysis and interpretation of the data, carried out molecular genetic studies and drafted the manuscript. GW contributed to the conception of the project and drafted the manuscript. BMD contributed to the conception and design of the project, analysis and interpretation of the data and drafted the manuscript. All authors have read and approved the final manuscript.
